# Brazilian Theraphosidae: a toxicological point of view

**DOI:** 10.1590/1678-9199-JVATITD-2021-0004

**Published:** 2021-11-22

**Authors:** Keven Wender Rodrigues Macedo, Lucas Jeferson de Lima Costa, Jéssica Oliveira de Souza, Isadora Alves de Vasconcelos, Jessica Schneider de Castro, Carlos José Correia de Santana, Ana Carolina Martins Magalhães, Mariana de Souza Castro, Osmindo Rodrigues Pires

**Affiliations:** 1 Laboratory of Toxinology, Department of Physiological Sciences, Institute of Biology, University of Brasília (UnB), Brasília, DF, Brazil.; 2 Laboratory of Biochemistry and Protein Chemistry, Department of Cell Biology, Institute of Biology, University of Brasília (UnB), Brasília, DF, Brazil.

**Keywords:** Brazilian Theraphosidae, Crude venom, Hemocytes, Biological active compounds, Spiders

## Abstract

The Theraphosidae family includes the largest number of species of the Mygalomorphae infraorder, with hundreds of species currently catalogued. However, there is a huge lack on physiologic and even ecologic information available, especially in Brazil, which is the most biodiverse country in the world. Over the years, spiders have been presented as a source of multiple biologically active compounds with basic roles, such as primary defense against pathogenic microorganisms or modulation of metabolic pathways and as specialized hunters. Spider venoms also evolved in order to enable the capture of prey by interaction with a diversity of molecular targets of interest, raising their pharmaceutical potential for the development of new drugs. Among the activities found in compounds isolated from venoms and hemocytes of Brazilian Theraphosidae there are antimicrobial, antifungal, antiparasitic and antitumoral, as well as properties related to proteinase action and neuromuscular blockage modulated by ionic voltage-gated channel interaction. These characteristics are present in different species from multiple genera, which is strong evidence of the important role in spider survival. The present review aims to compile the main results of studies from the last decades on Brazilian Theraphosidae with special focus on results obtained with the crude venom or compounds isolated from both venom and hemocytes, and their physiological and chemical characterization.

## Background

Mygalomorphae (Pocock, 1892) is an infraorder of spiders, which includes species from the family Theraphosidae, commonly known as tarantulas. These spiders are characterized by medium to large size, characteristic articulated chelicerae that move parallel to the axis of the animal's body, called orthognathic chelicerae. Despite their size inspiring fear, Theraphosidae usually are not dangerous to humans [1]. This family is the largest within Mygalomorphae, including 1004 species distributed in 152 genera [2]. The updated number of Theraphosidae in Brazil is not available, with the last record accounting 185 species divided in 36 genera [3]. 

The main source of compounds isolated from spiders comes from the venom and hemocytes. The tarantula venom is extracted through electrical stimulation at the base of the chelicerae, forcing its contraction and provoking the venom release. To obtain the hemocytes, the spiders are cooled then an apyrogenic needle is used to perform a cardiac puncture for hemolymph extraction. To avoid the coagulation or degranulation of the hemocytes, the extraction is conducted in the presence of sodium citrate buffer. The hemocytes are separated from plasma by centrifugation [4, 5, 6].

Tarantula venom has a complex composition, containing inorganic salts, nucleotides, free amino acids, polyamines, neurotransmitters, peptides and proteins. The venom generally acts on prey nervous system leading to paralysis due to the large number of neurotoxins as acylpolyamines and ion channel modifiers or pore-forming peptides [1, 7, 8].

The diversity of biological activities from crude venom, hemocyte extracts or compounds isolated from both sources, arouse the interest of the scientific community and industries. Antimicrobial, neurotoxic, cytotoxic, hemolytic and protease activities have been described throughout the years, and the molecules responsible for these activities have shown themselves to be promising in the development of new products like pesticides or pharmaceuticals [5, 9, 10].

In this review we present 43 studies performed with Brazilian Theraphosidae species along the last decades with focus on the structural and pharmacological characterization of biologically active compounds isolated from the venom or hemocytes of this family.

## Methods

### Search strategy

The selection of articles for this review was based on Preferred Reporting Items for Systematic Reviews and Meta Analyses (PRISMA) [11]. The search for articles was performed primarily in Google Scholar, followed by PubMed and ScienceDirect. The keywords for research were “Theraphosidae antimicrobial”, “Theraphosidae antitumoral” and specific researches each Theraphosidae species/genera selected once their occurrence in Brazil was check and confirmed based on World Spider Catalog (https://wsc.nmbe.ch/). Posteriorly were included “Theraphosidae venom”, “Theraphosidae toxins”, “Theraphosidae hemocytes”, “Theraphosidae venom composition” once new relevant activities described were included to the original antimicrobial/antitumoral focus of this review.

### Study selection and data extraction

The search on the 3 databases (Google Scholar = 1709; PubMed = 155; ScienceDirect = 572) resulted in a total of 2436, with 99 studies initially selected by title and abstract reading once they present information about venom and hemocytes compounds from Theraphosidae. References from studies with these inclusion criteria was check to include possible relevant articles. 

Sixty-seven articles were selected for full reading and papers without the focus on venom or hemocytes compounds, duplicated or unrelated information and focused on spiders that do not occur in Brazil were excluded after reading. Some studies with multiple spiders were selected when at least one of the species presented in the study showed the inclusion criteria, but only the spiders with inclusion criteria have their related results described. No boolean operator was utilized in these steps, all articles were selected by two authors with a third author evaluating the quality and eligibility of the studies.

The search in Uniprot (www.uniprot.org) for sequenced compounds were performed with the advanced search tool. Genera and species (sp. when species is unknown) was utilized as keywords on the database, the search results is presented on Table 1.

**Table 1.** Uniprot register of toxins extracted from Brazilian Theraphosidae spiders.

Uniprot entry	Entry name	Protein name	Source	Sequence	Reference
P83745	TX1_THEBL	κ-theraphotoxin-Tb1a	Theraphosa blondi venom	AACLGMFESCDPNNDKCCPNRECNRKHKWCKYKLW	Ebbinghaus *et al.* [15]
P83746	TX2_THEBL	κ-theraphotoxin-Tb1b	Theraphosa blondi venom	DDCLGMFSSCDPKNDKCCPNRVCRSRDQWCKYKLW	Ebbinghaus *et al*. [15]
P83747	TX3_THEBL	κ-theraphotoxin-Tb1c	Theraphosa blondi venom	DDCLGMFSSCDPNNDKCCPNRVCRVRDQWCKYKLW	Ebbinghaus *et al*. [15]
P82358	GOME_ACAGO	Gomesin	Acanthoscurria gomesiana hemocytes	QCRRLCYKQRCVTYCRGR	Silva *et al*. [21]
Q8I948	ACN1_ACAGO	Acanthoscurrin-1	Acanthoscurria gomesiana hemocytes	DVYKGGGGGRYGGGRYGGGGGYGGGLGGGGLGGGGLGGGKGLGGGGLGGGGLGGGGLGGGGLGGGKGLGGGGLGGGGLGGGGLGGGGLGGGKGLGGGGLGGGGLGGGRGGGYGGGGGYGGGYGGGYGGGKYK	Lorenzini *et al*. [24]
Q8I6R7	ACN2_ACAGO	Acanthoscurrin-2	Acanthoscurria gomesiana hemocytes	DVYKGGGGGRYGGGRYGGGGGYGGGLGGGGLGGGGLGGGKGLGGGGLGGGGLGGGGLGGGGLGGGKGLGGGGLGGGGLGGGGLGGGGLGGGKGLGGGGLGGGGLGGGRGGYGGGGYGGGYGGGYGGGKYK	Ferreira *et al*. [50]
P0DQJ3	TXA1_ACAGO	U1-theraphotoxin-Agm1a	Acanthoscurria gomesiana venom	IIECFFSCEIEKDGKSKEGKPCKPKGDKDKDKKCSGGWRCKIKMCLKI	Abreu *et al*. [20]
P0DQJ4	GEND1_ACAGO	U1-theraphotoxin-Agm2a	Acanthoscurria gomesiana venom	SCVHERETCSKVRGPLCCRGECICPIYGDCFCYGS	Abreu *et al*. [20]
P0DQJ5	VSTX1_ACAGO	U1-theraphotoxin-Agm3a	Acanthoscurria gomesiana venom	ACGSFMWKCSERLPCCQEYVCSPQWKWCQNP	Abreu *et al*. [20]
B3EWY4	TXAP1_ACAPA	U1-theraphotoxin-Ap1a	Acanthoscurria paulensis venom	IIECFFSCEIEKDGKSKEGKPCKPKGDKNKDKKCSGGWRCKIKMCLKI	Mourão *et al*. [31]
B3A0P0	TXAN1_ACANA	Mu-theraphotoxin-An1a	Acanthoscurria natalensis venom	IIECFFSCEIEKDGKSKEGKPCKPKGDKDKDKKCGGWRCKIKMCIKI	Rates *et al*. [35]
B3EWP8	RDNIN_ACARO	Rondonin	Acanthoscurria natalensis hemocytes	IIIQYEGHKH	Riciluca *et al*. [34]
B3EWQ0	JURTX_AVIJU	U-theraphotoxin Aju1a, (Juruin)	Avicularia juruensis venom	FTCAISCDIKVNGKPCKGSGEKKCSGGWSCKFNVCVKV	Ayroza *et al.* [42]
P0CC18	TXL1_LASSB	U1-theraphotoxin-Lsp1a, U1-TRTX-Lsp1a (LTx1)	Lasiodora sp. venom	FFECTFECDIKKEGKPCKPKGCKCKDKDNKDHKKCSGGWRCKLKLCLKF	Vieira *et al*. [46]
Q5Q114	TXLT2_LASSB	U1-theraphotoxin-Lsp1b (LTx2)	Lasiodora sp. venom	LFECTFECDIKKEGKPCKPKGCKCDDKDNKDHKKCSGGWRCKLKLCLKI	Vieira *et al*. [46]
Q5Q113	TXLT3_LASSB	U1-theraphotoxin-Lsp1c (LTx3)	Lasiodora sp. venom	FFECTFECDIKKEGKPCKPKGCKCDDKDNKDHKKCSGGWRCKLKLCLKF	Vieira *et al*. [46]
A3F7X1	TXLT4_LASSB	U2-theraphotoxin-Lsp1a, U2-TRTX-Lsp1a (LTx4)	Lasiodora sp. venom	CGGVDAPCDKDRPDCCSYAECLRPSGYGWWHGTYYCYRKRER	UniProtKB* [47]
A3F7X2	TXTR3_LASSB	U3-theraphotoxin-Lsp1a, U3-TRTX-Lsp1a (LTx5)	Lasiodora sp. venom	DDSLNKGEPCQFHCECRGASVLCEAVYGTRSPMYKCMIKRLPISVLDIMYQAERALEKLASSFRCE	UniProtKB* [47]
P0CC18	TXL1_LASPA	U1-theraphotoxin-Lp1a (LpTx1)	Lasiodora parahybana venom	FFECTFECDIKKEGKPCKPKGCKCKDKDNKDHKKCSGGWRCKLKLCLKF	Escoubas *et al*. [52]
P61506	TXL2_LASPA	U1-theraphotoxin-Lp1b (LpTx2)	Lasiodora parahybana venom	FFECTLECDIKKEGKPCKPKGCKCNDKDNKDHKKCSGGWRCKLKLCLKF	Escoubas *et al*. [52]

*Sequences registered under the title “Screening of *Lasiodora* sp. expression library and molecular cloning of *Lasiodora* sp. toxins in expression vectors” do not have any publication associated with the sequence registry.

## Results and Discussion

**Figure 1.**
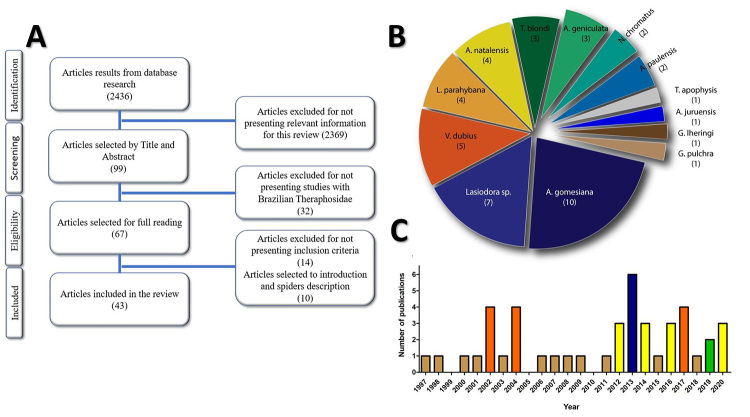
General results of research steps. **(A)** Article selection flow chart. **(B)** Number of articles selected for each species described. **(C)** Number of publications selected between 1997 and 2021.

The complete flow chart with the description of the selection process is presented in Figure 1A. The search resulted in 43 articles selected to inclusion on the review, contemplating thirteen species (Fig. 1B). The oldest publication dates from 1997 and newest from 2021 (Fig. 1C). Although some articles have been excluded to full description, they were utilized for introduction and spider description.

###  Theraphosa blondi 

**Figure 2.**
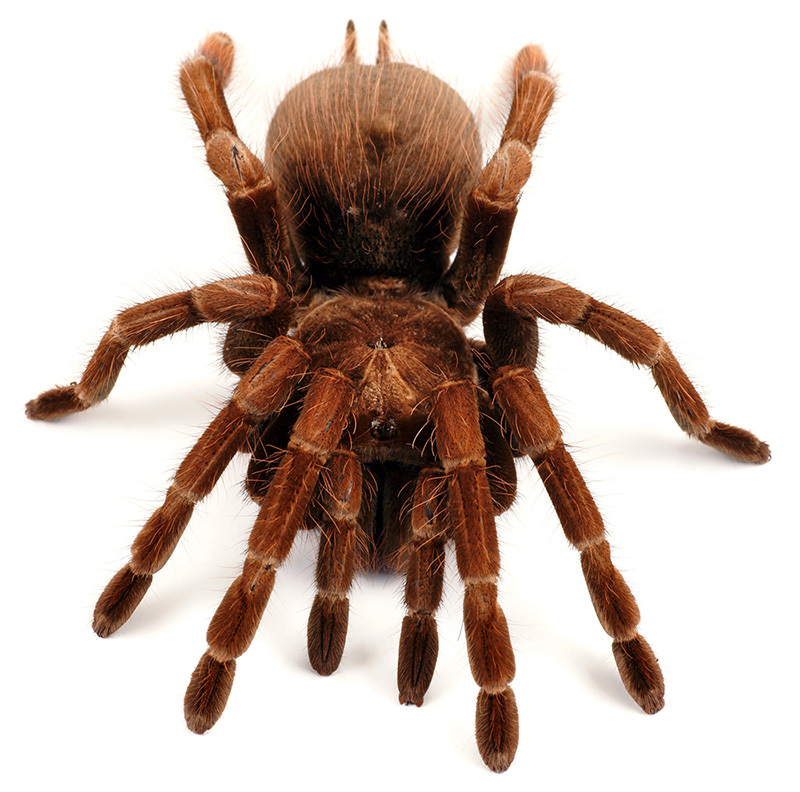
Specimen of Theraphosa blondi. Photo by Mirek Kijewski (ID 171295546).

 Theraphosa blondi (Latreille, 1804; Fig. 2) commonly known as Goliath bird eater spider, is one of the largest known spiders, both in size and mass with above 30 cm leg spam [12]. It occurs in the Amazon rainforest, can be found in northern Brazil, Suriname, Guyana, French Guiana, and southern Venezuela. *T. blondi* is a terrestrial spider and lives in deep burrows, usually found in marshy or swampy areas [12, 13].

In 2002, Fontana *et al*. [14] studied the mode of action of *T. blondi* venom in mouse phrenic nerve-diaphragm preparation. The venom caused partial and reversible neuromuscular blockage, not depressing spasms caused by direct stimulation or altering the membrane potential. The blockage caused by the venom suffered a weak antagonistic effect by neostigmine, which however, completely blocked the venom activity in miniature end plate potentials. The authors suggested the presence of toxins that interact with the terminal plaque receptor at the sites of acetylcholine as curare mimetic toxins, and toxins that inhibit the type P voltage-dependent calcium channel as an explanation for the different effects caused by the interaction of neostigmine with the venom [14].

In 2004, Ebbinghaus *et al*. [15] evaluated by whole-cell patch-clamp the effects of *T. blondi* (referred by the authors as *T. leblondi*) venom on voltage-dependent potassium (K_v_) channel-mediated currents. *T. blondi* venom inhibited A-type currents in recombinant Kv4.2 channels expressed by cultured hippocampal neurons from C57/Bl6 mice (*Mus musculus*) and HEK 293 cells, presenting selective activity in both cases. The venom was also tested on recombinant Kv1.3, Kv1.4, Kv2.1, and Kv3.4 channels also expressed in HEK 293 cells; however, the venom did not show effect against them [15]. 

The authors purified the venom by Reverse Phase - High Performance Liquid Chromatography (RP-HPLC) and sequenced three 35 amino acid peptides named as TLTx1, TLTx2 and TLTx3 by tandem mass spectrometry. TLTx1 caused inhibition in recombinant Kv4.2 channels (IC_50_ = 200 nM), and slowed Kv4.2 activation kinetics. The venom also slowed the inactivation caused by macroscopic current. The authors suggested that TLTx1 may act as a Kv4.2 channel gating modifier [15].

Also in 2004, *T. blondi* venom was characterized through mass fingerprinting using several mass spectrometric methods, including matrix-assisted laser desorption/ionization time-of-flight mass spectrometry (MALDI-TOF/MS), on-line liquid chromatography/electrospray ionization mass spectrometry (LC/ESI-MS), and nanospray ionization/hybrid quadrupole time-of-flight mass spectrometry (nanoESI-QqTOFMS). Direct nanoESI-QqTOF-MS and MS/MS experiments were considered very efficient methods for peptidomic analysis of the crude *T. blondi* venom, and the best performance was obtained using nanoESIQqTOF-MS, which detected 65 molecules with high mass accuracy [16]. 

Three major peptides that inhibit voltage-gated potassium channels were selected as *T. blondi* venom biomarkers: TlTx1, TlTx2 and TlTx3. These peptides were obtained by RP-HPLC and cleaved by trypsin, Asp-N and Glu-C endoproteinases to generate shorter fragments suitable for MS/MS experiments using a combination of nanoESI-MS/MS and MS/MS, all peptide sequences were also confirmed by Edman degradation [16].

Nowadays TlTx1, TlTx2 and TlTx3 are registered in Uniprot database κ-theraphotoxin-Tb1a, κ-theraphotoxin-Tb1b and κ-theraphotoxin-Tb1c, respectively, as seen in Table 1. 

##  Theraphosa apophysis 

*T. apophysis* (Tinter 1991), known as pink foot goliath tarantula, is another giant tarantula belonging to *Theraphosa* genus, with leg span up to 30 cm. It occurs in Brazil, Colombia and Venezuela [2, 17].

Two peptides with inhibitory activity on sodium and calcium voltage-gated channels (Na_v_ and Ca_v_) were isolated by Cardoso *et al*. [18] from *T. apophysis* venom. Both compounds present high affinity with Na_v_ 1.2, Na_v_ 1.7, and Ca_v_ 3.1 channels; low affinity with Na_v_ 1.4, Na_v_ 1.5 channels. The potency against Na_v_ 1.6 channels was lower than observed in Na_v_ 1.7 channels. These new peptides, named as theraphotoxin-Tap1a and theraphotoxin-Tap2a (TRTX-Tap1a and TRTX-Tap2a) were isolated from the crude venom by RP-HPLC followed by alkylation and reduction. The molecular masses of 4179.5 (Tap1a) and 3843.4 (Tap2a) Da were obtained by MALDI-TOF/MS and Edman degradation revealed sequences of 35 and 33 amino acids respectively. Recombinants of both peptides were produced by *E. coli* periplasmatic expression system [18].

The recombinants activities were tested by whole-cell patch clamp against the channels expressed by human HEK293 cells with rTap1a showing to be more potent than rTap2a. Using male C57BL/6J mice (*M. musculus*) with irritable bowel syndrome a 10 µM dose reduced the mechanic sensitivity of bladder, reduced nociceptive response *ex vivo* and visceral pain *in vivo*. The authors concluded that the combination of Na_v_ and Ca_v_3 inhibition presents great potential in treatment of chronic visceral pain [18].

##  Acanthoscurria gomesiana 

**Figure 3.**
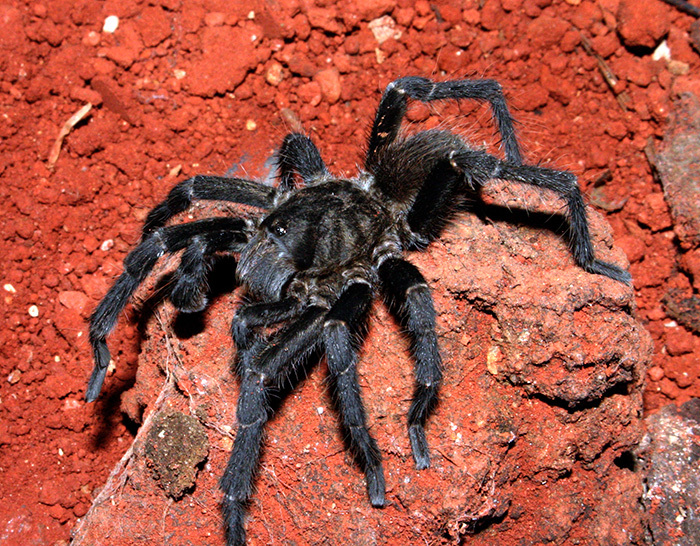
Male specimen of *Acanthoscurria gomesiana.* Collection of arachnids from the Department of Zoology, University of Brasília, no. 3281. Photo by João de Jesus Martins.

 Acanthoscurria gomesiana (Mello-Leitão, 1923. Theraphosidae, Mygalomorphae; Fig. 3), commonly known as São Paulo Black Tarantula, has approximately 5 cm, and is distributed in the south of Minas Gerais and northeast region of São Paulo. In nature, it can be found in natural holes, termite mounds, vicinity of roots and under rotten trunks [19].

Abreu *et al.* [20] investigated the complete peptidome of *A. gomesiana* venom. The peptide fraction, obtained by Solid-Phase Extraction, showed antimicrobial activity against Gram-negatives *E. coli* SBS363 (MIC could not be obtained among the tested concentrations), *E. cloacae* β12 (MIC between 22 and 45 ng/µL) and against yeast *C. albicans* MDM8 (MIC between 11 and 22 ng/µL) [20].

The native peptides from the venom were analyzed by Ultra Definition Mass Spectrometry^E^ (UDMS^E^) to determine the precursor masses and allow the sequencing with the fragmented ions [20]. 

The peptide fraction was digested with multiple enzymes (trypsin/Lis-C, chymotrypsin, Glu-C and thermolysin) and the fragments were analyzed by LC-MS/MS. 135 peptides were found from the digestions, resulting in 17 proteins including three new theraphotoxins: (Table 1): U1-TRTX-Agm1a, which has a single aspartate (position 29) different from *A. paulensis* U1-TRTX-Ap1; U1-TRTX-Agm2a, which derives from *A. geniculata* genicutoxin-D1 precursor and U1-TRTX-Agm3 [20].

Gomesin was the first antimicrobial reported from spider hemocytes. It was isolated and characterized in 2000 by Silva *et al* [21]. The hemolymph was centrifuged in presence of sodium citrate buffer and the hemocytes were separated, washed in the same buffer and lysed in vacuum centrifuge. The lysed hemocytes compounds were subjected to solid phase extraction, eluted in 40% acetonitrile in acidified water, and then concentrated in vacuum centrifuge. The resultant fraction was purified by RP-HPLC resulting in three antimicrobial fractions (AGH1, AGH2, and AGH3). AGH2 fraction was analyzed by MALDI-TOF/MS and ESI-MS analysis indicating a molecular mass of 2270.4 Da, and Edman degradation sequencing resulted in an 18 amino acids sequence, presented in Table 1, named as gomesin [21].

Gomesin has a pyroglutamic acid in N-terminal portion and an amidated arginine in C-terminal portion, presenting two disulfide bonds. The authors also described that gomesin has similarities with the antimicrobial peptides isolated from horseshoe crabs (*Tachypleus tridentatus*) tachyplesins and polyphemusins (50% of similarity in both cases), androctonin isolated from the Sahara scorpion (*Androctonus australis*) and protegrin-1, isolated from leukocytes of porcine (*Sus scrofa*), presented 23% and 17% of similarity, respectively. All these peptides have two disulfide bonds formed by cysteines 1-4 and 2-3 in their structures [21].

Gomesin showed activity against a wide spectrum of microorganisms including Gram-positives (MICs between 0.2 and 12.5 µM), Gram-negatives (MICs between 0.4 and 6.25 µM), Filamentous fungi (MICs between 0.4 and 25 µM) and yeasts (MICs between 0.15 and 25 µM) as seen in Table 2. It also reduced the viability of *Leishmania amazonensis* promastigotes in viability assay (IC_50_= 2.5 µM), as seen in Table 2. Gomesin also showed hemolytic activity against human erythrocytes, with range between 7% and 22% in concentrations from 1 µM to 100 µM. At low concentrations (0.1 and 0.2 µM) gomesin caused less than 5% hemolysis [21]. 

**Table 2.** Minimal inhibition concentration of toxins extracted from Brazilian Theraphosidae spiders with confirmed antimicrobial activities.

Toxin MICs								
Microorganism	Gomesin	Acanthoscurrins	Lasiodora crude venom	Mygalin/ MygAgNPs	Rondonin	Juruin	EiLAH	VdTX-1
Aerococcus viridans	0.8-1.6 µM	n.t	n.t	n.t	n.t	n.t	n.t	n.t
***Aeromonas* sp*.***	n.t	n.t	62.5 µg/mL	n.t	n.t	n.t	n.t	n.t
Agrobacterium tumefaciens	n.v	n.t	n.t	n.t	n.t	n.t	n.t	n.t
Alcaligenes faecalis	>100 µM	n.t	n.t	n.t	n.t	n.t	n.t	n.t
Alternaria brassicola	0.4-0.8 µM	n.t	n.t	n.t	n.t	n.t	n.t	n.t
Aspergilus fumigatus	1.6-3.15 µM	n.t	n.t	n.t	n.t	n.t	n.t	n.t
Aspergilus niger	n.t	n.t	n.t	n.t	n.t	5-10 µM	n.t	n.v
Bacillus cereus	6.25-12.5 µM	n.t	n.t	n.t	n.t	n.t	n.t	n.t
Bacillus megaterium	0.2-0.4 µM	n.t	n.t	n.t	n.t	n.t	n.t	n.t
Bacillus subtilis	n.t	n.t	62.5 µg/mL	n.t	n.t	n.t	n.v	n.t
Bacillus thuringiensis	1.6-3.15 µM	n.t	n.t	n.t	n.t	n.t	n.t	n.t
Bauveria bassiana	12.5-25 µM	n.t	n.t	n.t	n.v	n.v	n.t	n.t
** Candida albicans IOC 45588**	0.15-0.3 µM	1.15-2.3 µM	125 µg/mL	n.v	16.5 μM	2.5-5 μM	n.t	n.t
** Candida albicans MDM8**	n.t	n.t	n.t	n.v	16.5 μM	2.5-5 μM	n.t	n.t
Candida guillermondii	n.t	n.t	n.t	n.t	33.5 μM	2.5-5 μM	n.t	6.25-12.5 μM
Candida glabrata	12.5-25 µM	n.t	n.t	n.t	16.5 μM	2.5-5 μM	n.t	6.25-12.5 μM
Candida krusei	n.t	n.t	7.8 µg/mL	n.t	33.5 μM	2.5-5 μM	n.t	6.25-12.5 μM
Candida parapsilosis	n.t	n.t	31.25 µg/mL	n.t	33.5 μM	2.5-5 μM	n.t	n.v
Candida tropicalis	3.15-6.25 µM	n.t	3.9 µg/mL	n.t	16.5 μM	2.5-5 μM	n.t	6.25-12.5 μM
** Cladosporium sp **	n.t	n.t	n.t	n.t	n.t	n.t	n.t	6.25-12.5 μM
Cryptococcus neoformans	0.8-1.6 µM	n.t	n.t	n.t	n.t	n.t	n.t	n.t
Enterobacter cloacae β12	3.15-6.25 µM	n.t	n.t	n.t	n.t	n.t	n.t	50 μM
Enterococcus faecalis	6.2-12.5 µM	n.t	n.t	n.t	n.t	n.t	227.5 µg/mL	n.t
Erwinia carolovora calorovora	3.15-6.25 µM	n.t	n.t	n.t	n.t	n.t	n.t	n.t
** Escherichia coli 1106**	0.8-1.6 µM	n.t	n.t	n.t	n.t	n.t	n.t	n.t
Escherichia coli ATCC 25922	n.t	n.t	n.t	n.t	n.v	n.v	n.v	6.25-12.5 μM
** Escherichia coli D22**	0.4-0.8 µM	n.t	n.t	n.t	n.t	n.t	n.t	n.t
** Escherichia coli D31**	0.8-1.6 µM	2.3-5.6 µM	n.t	n.t	n.t	n.t	n.t	6.25-12.5 μM
** Escherichia coli SBS363**	0.4-0.8 µM	n.t	n.t	85 µM/ 19-58 nM	n.v	n.v	n.t	n.t
Fusarium culmorum	0.4-0.8 µM	n.t	n.t	n.t	n.t	n.t	n.t	n.t
Fusarium oxysporum	0.4-0.8 µM	n.t	n.t	n.t	n.t	n.t	n.t	n.t
Klebsiella pneumoniae	3.15-6.25 µM	n.t	15.62 µg/mL	n.t	n.t	n.t	n.v	n.t
Leishmania amazonenses	2.5 µM	n.t	n.t	n.t	n.t	n.t	n.t	n.t
Listeria monocytogenes	0.8-1.6 µM	n.t	n.t	n.t	n.t	n.t	n.t	n.t
Micrococcus luteus	0.4-0.8 µM	>5.6 µM	7.8 µg/mL	n.v	n.v	n.v	n.t	6.25-12.5 μM
Nectria haematococca	0.2-0.4 µM	n.t	n.t	n.t	n.t	n.t	n.t	n.t
Neurospora crassa	0.4-0.8 µM	n.t	n.t	n.t	n.t	n.t	n.t	n.t
Nocardia asteroids	1.6-3.15 µM	n.t	n.t	n.t	n.t	n.t	n.t	n.t
Pediococcus acidolacrici	3.15-6.25 µM	n.t	n.t	n.t	n.t	n.t	n.t	n.t
Pseudomonas aeruginosa	1.6-3.15 µM	n.t	31.25 µg/mL	n.t	n.v	n.v	n.t	n.t
Saccharomyces cerevisiae	1.6-3.15 µM	n.t	n.t	n.t	n.t	n.t	n.t	n.t
Salmonella thyphinurium	0.8-1.6 µM	n.t	n.t	n.t	n.t	n.t	n.t	n.t
** Serralia marcescens Db11**	n.v	n.t	n.t	n.t	n.t	n.t	n.t	n.t
Staphylococcus aureus	1.6-3.15 µM	n.t	7.81 µg/mL	n.t	n.v	n.v	n.v	6.25-12.5 μM
Staphylococcus epidermidis	0.8-1.6 µM	n.t	n.t	n.t	n.v	n.v	n.t	6.25-12.5 μM
Staphylococcus haemolyticus	0.8-1.6 µM	n.t	n.t	n.t	n.t	n.t	n.t	n.t
Staphylococcus saprophyticus	0.8-1.6 µM	n.t	n.t	n.t	n.t	n.t	n.t	n.t
Streptococcus pyogenes	1.6-3.15 µM	n.t	n.t	n.t	n.t	n.t	n.t	n.t
Tricoderma viridae	0.4-0.8 µM	n.t	n.t	n.t	n.t	n.t	n.t	n.t
Tricophyton mentagrophytes	0.8-1.6 µM	n.t	n.t	n.t	n.t	n.t	n.t	n.t
Trichosporium sp.	n.t	n.t	n.t	n.t	n.t	n.t	n.t	6.25-12.5 μM
Trichosporon sp.	n.t	n.t	n.t	n.t	1.1 μM	n.t	n.t	n.t
Xhantomonas campestris pv. Orizae	3.15-6.25 µM	n.t	n.t	n.t	n.t	n.t	n.t	n.t

n.v: no value related; n.t: not tested

The three-dimensional structure of gomesin was elucidated by two-dimensional nuclear magnetic resonance (2D-NMR) followed by calculation of the molecular dynamics [22]. Gomesin exhibiting a hairpin-like structure folded in two antiparallel β-sheets (pGlu1-Tyr7 and Arg10-Arg16), with a non-canonical β-turn connecting both strands. Gomesin structure was also compared to the antimicrobials protegrin-1 (*S. scrofa*) and androctonin (*A. australis*), showing similarities in the distribution of the hydrophilic and hydrophobic residues, so it was suggested that the membrane interaction occurs in similar manner [22].

Gomesin also showed activity against melanoma cells, as described by Ikonomopoulou *et al*. [23]. The group compared the antiproliferative activity of gomesin (referred here as AgGom) and a gomesin-like homologous (HiGom) from the Australian spider *Hadronyche infensa* against murine melanoma MM96L cells with mutation in BRAF genes and normal human neonatal foreskin fibroblasts (NFF) cell line. Authors concluded that both peptides cause late apoptosis in dose-dependent manner against MM96L cells, reducing both viability and proliferation (AgGom IC_50_= 25 µg/mL; HiGom IC_50_= 6.3 µg/mL), but no activity against NFF cells was observed in concentrations < 50 µg/mL. Both molecules also reduced proliferative and metastatic capacity in zebrafish (*Danio rerio*) AVATAR MM96L xenograft tumor models. AgGom and HiGom act on the cells via activation of the p53/p21 checkpoint and Hippo pathway. AgGom and HiGom also inhibit the MAP kinase pathway. The activation cascades caused by AgGom and HiGom stimulate the accumulation of reactive oxygen species (ROS), reducing the membrane potential of the mitochondria that results in the late cell apoptosis [23]. 

Acanthoscurrins are glycine-rich antimicrobial peptides isolated from *A. gomesiana* hemocytes [24]. The hemocytes were treated in the same manner described by [21] during the purification of the gomesin, and the fractions AGH1, AGH2 and AGH3 were obtained. Mass spectrometry showed that AGH2 corresponds to gomesin. This study focused on AGH3, which was purified by RP-HPLC and characterized ESI-MS/MS. Capillary electrophoresis confirmed the presence of two molecules with similar molecular masses (10,225 Da and 10,111 Da) [24].

Edman degradation and cDNA cloning confirmed two isoforms with 132 and 130 amino acids (about 73% glycine residues), named as acanthoscurrin-1 and acanthoscurrin-2 (Table 1), respectively. The only difference between both peptides is the absence of two glycine in acanthoscurrin-2. The authors described their primary structures as unique, once they did not show structural similarities with the glycine-rich antimicrobial peptides isolated from insect larvae as AFP (*Sarcophaga peregrina*), holotricin-3 (*Holotrichia diomphalia*), and tenecin-3 (*Tenebrio molitor*), or isolated from Brassicaceae (*Capsella bursa-pastoris*) as the shepherins [24]. 

Antimicrobial tests were performed on *Microccus luteus* (no activity reported using concentration up to 5.6 µM), *Escherichia coli* (MIC = 2.3 - 5.6 µM) and *Candida albicans* (MIC = 1.15 - 2.3 µM) as seen in Table 2 [24].

The third antimicrobial compound (AGH1) present in *A. gomesiana* hemocytes is an acylpolyamine, named as mygalin, characterized by Pereira *et al*. [25]. Authors identified mygalin between three different RP-HPLC fractions with antimicrobial activity, two of them corresponding to the previously described gomesin and acanthoscurrins. Mygalin complete purification was achieved by an additional size exclusion chromatography step. ESI-MS revealed that mygalin has 417.3 Da [25].

**Figure 4.**
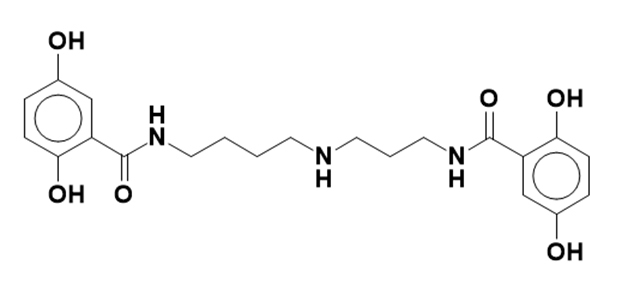
Mygalin structure.

The structure of mygalin (Fig. 4) was elucidated by tandem mass spectrometry (MS/MS) and two spectroscopic techniques, Nuclear Magnetic Resonance (NMR) and Ultraviolet (UV) Spectroscopy, identified mygalin as bis-acylpolyamine N1N8-bis(2,5-dihydroxybenzoyl)spermidine [25].

Mygalin antimicrobial activity (Table 2) was tested against *E. coli*, *M. luteus*, and *C. albicans*, showing activity only against *E. coli* (MIC = 85 µM). However, the activity of mygalin (2.6 to 170 µM) against *E. coli* was inhibited in catalase presence (100 µg/mL), so the authors concluded that antimicrobial mechanism involves the H_2_O_2_ production. Interestingly, the activity of mygalin against *E. coli* was 4-fold higher (MIC = 21.2 µM) when the culture medium was supplemented with a trace elements solution. [25]. 

Mygalin was also described as having an immunomodulatory effect by Mafra *et al*. [26]. The toxicity of mygalin (5 to 40 µg/mL) against splenocytes and macrophages collected from euthanized C57BL/6 mice was evaluated by MTT assay, which showed that mygalin did not reduce the cells viability. Mygalin activates the enzyme inducible nitric oxide synthase (iNOS) enhancing the production of nitrite and inducing the TNF-α production by macrophages. _L_-NIL, a specific iNOS inhibitor, presence ceased the nitrite production by macrophage suggesting that the nitrite production occurs independently from exogenous IFN-γ. Mygalin did not have direct action on the inflammasome complexes, once it did not induce IL-1β secretion or activate the caspase-1. Authors suggested that mygalin target are T cells once it stimulate the production of IFN-γ, a Th1 cytokine, but does not cause the production of Th2 cytokines as IL-5 [26]. 

Mygalin showed anticonvulsant activity which was reported in 2013 [27], preventing seizures provoked in male Wistar rats (*Rattus norvegicus*) by N-methyl-D-aspartate (NMDA) and pentylenetetrazole (PTZ). Mygalin (2 µg/µL) presented anticonvulsant activity of 16.6 % against seizures induced PTZ. The NMDA experiment revealed a reversed dose dependence curve, a 2ng/µL dose caused reduction of 83.3% in the seizures. To evaluate possible side effects, the rats were submitted to Open field, Rotarod and Morris Water Maze tests to analysis of locomotor activity, motor impairment and neurological disorders. Mygalin treated rats presented mild behavioral changes in comparison to rats treated with conventional anticonvulsant drugs. Authors hypothesized that mygalin may be an antagonist to NMDA receptor [27].

Mygalin was subjected to multiple tests to elucidate the mechanisms responsible for the antimicrobial activity against *E. coli*. In a viability assay, mygalin (0.5 mM) was more effective than H_2_O_2_ (1 mM) [28]. Alkaline electrophoresis gel showed that mygalin causes oxidative DNA damage, which was also observed in *E. coli* model by confocal microscopy. This result was supported by DNA-DAPI fluorescence assay. DAPI is a fluorescent dye that intercalates with DNA double-helix and is commonly used to evaluate structural damage on DNA. Filamentation assay with 10^6^ CFU/mL treated with 0.5 mM for 3h revealed the capacity of mygalin to interfere on cell division by binding to DNA, causing inhibition of its synthesis *in vivo* [28]. 

Mygalin (0.5 mM) also showed the capacity to disrupt cells membrane, which was evaluated by propidium iodide (PI) uptake combined with esterase activity assays. The esterase was stained with CFDA and variation of membrane permeability to PI was confirmed by Confocal Microscopy. Mygalin (0.5 mM) also shows contribution to formation of ROS, which is higher than observed in the H_2_O_2_ controls (0.25, 0.5 and 1-mM doses). Authors suggested that ROS production as one of the main mechanisms behind the DNA damage [28].

Mygalin interaction with LPS was confirmed by spectrometric evaluation of free mygalin when exposed to LPS from an initial 0.5 mM concentration. Finally, mygalin was also confirmed as a Fe^+2^ chelator when it in a dose-dependent manner (0 - 1000 µM) reduced the dihydrorhodamine hydrochloride (DHR) oxidation [28]. 

Recently, the antimicrobial and antitumoral activities of seven mygalin silver nanoparticles (MygAgNPs) were evaluated by [29]. The MygAgNPs were synthetized by photoreduction method, forming spherical particles with sizes from 10 to 60 nm tested against *E. coli*, revealing a reasonable enhance in the antimicrobial activity (MICs between 19 and 58 nM according to mygalin concentration used to nanoparticle synthesis) when compared to the peptide native (MIC = 1 mM) form [29].

The nanoparticle named as MygAgNP1 activity was evaluated against MCF-7 cells and normal NHI/3T3 murine fibroblast (ATCC^®^CRL-1658) in doses from 2.5 to 80 µL of nanoparticles. Authors highlighted the 5 µL dose, which caused death of approximately 52% of the tumoral cells, but only 13% of the NHI/3T3 cells [29].

## Acanthoscurria paulensis

**Figure 5.**
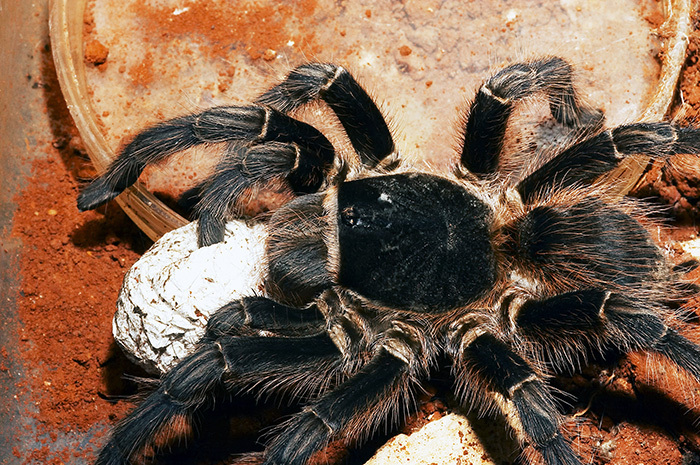
Female specimen of *Acanthoscurria paulensis.* Collection of Arachnids from the Department of Zoology, University of Brasília, no. 3423. Photo by João de Jesus Martins.

Distributed in all the central region of Brazil and in the states of Mato Grosso, Goiás, Minas Gerais, Mato Grosso do Sul, Paraná and Rio Grande do Sul, *Acanthoscurria paulensis* (Mello-Leitão, 1923; Fig. 5) is a big brownish mygalomorph, usually found inside *Armitemes* sp. termite mounds [30].

Mourão *et al*. [31] characterized the pharmacological activities of the Brazilian tarantula *A. paulensis* venom. A MALDI/TOF screening of 60 fractions obtained through fractionation by RP-HPLC showed a total of 97 components ranging from 600 to 22,000 Da. The molecular ions 601.4 and 728.6 Da, observed in mass spectra, were suggested as acylpolyamines corresponding to ions originally discovered in the tarantula *Aphonopelma chalcodes* venom and further described in *Lasiodora parahybana* venom [31]. 

A 20 µg/g *A. paulensis* venom dose was injected intraperitoneally in Swiss albino mice (*Mus musculus*), causing hypoactivity, prostration, contortion, dyspnea, ataxia and constipation. When the dose was increased to 25-30 µg/g, abdominal spasm, anuria and general flaccid paralysis were observed, with a death rate of 60 to 80%. Using 40 µg/g of venom, all individuals presented convulsions after cyanosis, tachycardia and spams. The 40 µg/g dose also resulted in death of all animals, which occurred approximately 90 minutes after the venom administration. The lethal dose for killing 50% of the mice (LD_50_) was 25.4 ± 2.4 µg/g. Mice utilized to lethality assay were dissected, and the organs were fixed in formalin, and embedded in paraffin. Histological sections stained with hematoxylin-eosin showed no alteration in the heart, lung, kidney, liver, or spleen. The authors also attested that no nociceptive behavior was induced in concentrations up to 20 µg/mice hind-paw [31].

The edematogenic activity of *A. paulensis* venom was tested with subplantar injection (20, 40 and 60 µg of venom/paw) in the hind paw of Wistar rats (*Rattus norvegicus*). The authors observed significant differences in the edema formation caused by each dose, especially when compared the lower (about 20% edema after 2 hours) and higher doses (about 50% edema after 2 hours) [31].

Cardiotoxicity assays were performed with the frog *Lithobates catesbeianus,* using *in situ* heart and isolated ventricular slices. For this assay, it was used the crude venom (50 µg), Low Molecular Mass Fraction (LMMF, 12.5 µg), and Protein Fraction (PF, 50 µg). The crude venom and LMMF caused cardiac arrest, but the activity was inhibited by atropine (2 µg), suggesting that the effect depends on acetylcholine receptors activation [31].

In the same year Mourão *et al*. [32] also identified and characterized a 48 amino acids peptide toxin from *A. paulensis* venom, named as Ap1a, which presents moderate similarity (67%) with the Huwentoxin-II isolated from the spider *Haplopelma schmidti*. Ap1a was purified by RP-HPLC, exhibiting a molecular mass of 5457.79 Da according to MALDI-TOF/MS mass spectrometry analysis. This peptide was reduced, alkylated and digested by Glu-C endopeptidase, and fragments were sequenced using Edman degradation and MS/MS, resulting in a partial sequence of 38 amino acid residues and three disulfide bonds formed by linkage of the cysteines 1-4; 2-5; 3-6. The full peptide sequence (Table 1) was obtained by transcriptomic analysis [32]. 

The authors reported that Ap1a causes dose-dependent paralysis on *Spodoptera frugiperda* larvae by intraperitoneal injection (ED_50_ = 13.01 ± 4.21 µg/g). It also interferes on frequency and amplitude of *Drosophila melanogaster* Giant Fiber Tergo Trochanteral Motor neurons (GF-TTM) and Dorsal Longitudinal Motor neurons (GF-DLM), dose-dependently reducing responses to electro stimulation at 100 Hz in both neurons when doses between 0.21 pM/g and 4.18 pM/g were used. The responses to direct stimulation stopped after 15 minutes in all the tested concentrations [32].

Ap1a was applied in a single dose in Swiss albino mice (*M. musculus,* 30 µg/ animal). After 10 minutes the toxin provoked urination, myoclonus and hypermobility, and animals presented generalized seizures after 12 minutes that led to death by respiratory failure 25 to 35 minutes after application [32].

Electrophysiological assays were performed using Ap1a at 1 μM, however it did not cause any stimulation in nicotine receptors expressed by rhabdomyosarcoma TE671 cells (ATCC® HTB-139). Human sodium-gated channels hNa_v_1.2, hNa_v_1.4, hNa_v_1.5 and hNa_v_1.6 also did not present significative effects caused by Ap1a [32]. 

## Acanthoscurria natalensis

**Figure 6.**
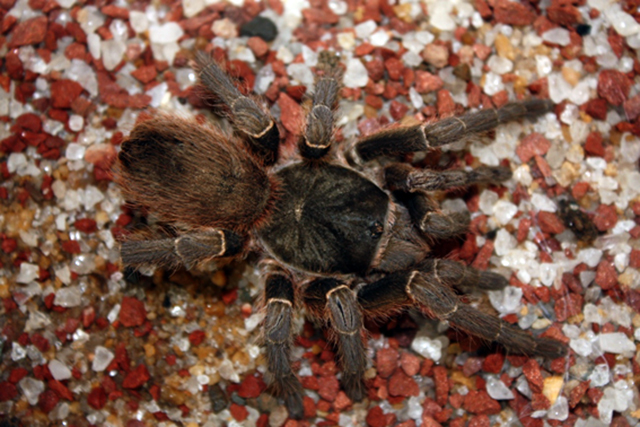
*Acanthoscurria natalensis* collected in Goiás state (Monte Alegre city). Photo by Osmindo R. Pires Jr.

*A. natalensis* (Chamberlin, 1917; Fig. 6), commonly known as Natal Brown Bird eater, is a species of tarantula that occurs in the Brazilian biomes Caatinga and Cerrado. It is a non-aggressive species with wide distribution among the Brazilian States [33]. *A. rondoniae* (Mello-Leitão, 1923) is synonym for *A. natalensis* [2, 33].

Rondonin is an antifungal peptide isolated from *A. natalensis* (cited by authors as *A. rondoniae*) hemolymph by RP-HPLC [34]. The molecular mass of 1236.776 Da was obtained by MALDI-TOF/MS and the 10 amino acids sequence (Table 1) was obtained by *de novo* analysis by liquid chromatography mass spectrometry (LC/MS) [34].

Although testes for bacteria, yeast, and fungi, rondonin only caused growth inhibition in *Candida* spp*.* (MICs between 16.5 and 33.5 μM) and *Trichosporon* sp. (MIC = 2.1 μM) as seen in Table 2. It also did not show toxicity to human erythrocytes with 0% hemolysis in concentrations up to 134 μM. The results suggested that rondonin antifungal properties may be specific against yeasts [34].

Rates *et al*. [35] isolated, characterized primary structure and determined electrophysiological effects of the anti-insect peptide µ-theraphotoxin-An1a (µ-TRTX-An1a) from the *A. natalensis* venom using two dimensional (cation exchange followed by RP-HPLC) or one-dimensional chromatography (RP-HPLC). A 37 N- terminal amino acid sequence (Table 1) was obtained by Edman degradation and complemented by *tandem* mass spectroscopy with LTQ Orbitrap, resulting in a 47 amino acid sequence. MALDI-TOF/MS analysis revealed that µ-TRTX An1a has a molecular mass of 5370.67 Da. The sequence of µ-TRTX-An1a showed similarities with U1-TRTX-Hh1a, previously known as huwentoxin-II, from *Haplopelma huwenum* [35].

The electrophysiological experiments were conducted in cockroach (*Periplaneta americana*) Dorsal Unpaired Median Neurons (DUM neurons). A 100 nM dose of µ-TRTX-An1a induced membrane depolarization, increased spontaneous firing frequency and reduced the action potential amplitude peaks. The toxin produced an increase in the frequency of action potential discharge associated slight depolarization, increasing the frequency of spontaneous firing (after 15 minutes of the toxin administration), resulting in a total disappearance the potential action 20 minutes after the exposure [35].

The test with the whole-cell under voltage clump condition, µ-TRTXAn1a (100 nM) promoted a partial blockade of the voltage-dependent sodium current amplitude in DUM neurons, without affecting its voltage dependence [35].

Authors correlated the blockage of Na current with the reduction in the spontaneous action potential amplitudes. They also suggested that µ-TRTX-An1a affects voltage-dependent sodium channels in insects’ neurons, which are possibly one of the channels targeted by this toxin [35].

In 2019, Barth *et al*. [36] isolated and characterized a protein complex formed by a hyaluronidase and a cysteine-rich secretory (CRISP)-like protein from *A. natalensis* venom. The crude venom was purified by RP-HPLC, and a fraction with hyaluronidase activity presented 53 kDa monomer and oligomers of 124 and 178 kDa were obtained by 1D Blue Native PolyAcrylamide Gel Electrophoresis (BN-PAGE). A 2D BN/SDS-PAGE revealed the presence of two subunits: a portion with hyaluronidase activity and 53 kDa named AnHyalH and a CRISP-like subunit with 44 kDa, named AnHyalC. Both subunits were sequenced by Edman degradation and compared with databases by Blast homology searches. AnHyalH showed 67% of similarity with the hyaluronidase from *Brachypelma vagans,* while AnHyalC presented 82% coverage with *Grammostola rosea* CRISP-like protein. The authors suggested that the CRISP protein present in the complex possibly contributes with AnHyal enzymatic activity [36].

In 2020, a multiomic study (venom gland transcriptomic, venom proteomic and peptidomic) was performed with *A. natalensis* (referred by authors as *A. rondoniae*) to present this approach as a viable method to identify and characterize peptides while investigating antimicrobial, antiviral and antitumoral activities *in silico* [37]. The venom glands were extracted, and the cDNA library obtained by TruSeq RNA Sample Prep Kit protocol followed by *de novo* assembly to eliminate redundancies, resulting in 92,889 transcripts [37].

To perform the proteomic and peptidomic analysis both the crude venom and aliquots digested with trypsin (for both analysis), Asp-N, Glu-C, chymotrypsin and thermolysin (only for peptidomic) were reduced and alkylated prior to analysis by nano flow LC-MS/MS. The combination of both techniques resulted in 18 toxins fully sequenced, quantified and validated: 11 Cysteine Rich Proteins, with one characterized as the U1-TRTX-Agm3a isolated from *A. gomesiana* venom and 10 new CRPs; and 7 peptides shorter than 10 amino acids [37]. 

The *in silico* predictions were realized with the CRPs revealing high antibacterial (scores from 0.843 to 0.997), antifungal (0.579 - 0.989) and antiviral (0.696 - 0.968) potentials with the U1-TRTX-Agm3a and a CRP named as U1-TRTX-Ar1b also revealing antitumoral potential tested by Random Forest and Support Vector Machine Anticancer Peptide Methods. The short peptides present lower antimicrobial and antiviral potentials than the CRPs, however 4 of them (sequences: PLPVFV, VPPILKY, VVVPFVV and VLPPLKF) present scores above 0.5 in both methods and have been characterized as potential anticancer peptides [37].

## Acanthoscurria geniculata

*A. geniculata* (Koch, 1941), commonly known as Brazilian White-knee Tarantula. Found in the Brazilian states of Rondônia, Roraima, Pará, and Mato Grosso, and identified by its coloration pattern with pink setae on legs and at the front border of the carapace [2, 38].

Sanggaard *et al*. [39] realized a genomic study with *A. geniculata* (Mygalomorph model) and *Stedodyphus mimosarum* (Araneomorph model) to determine the influence of predation methods in the composition of venom, silk and digestive fluids produced by these species [39]. 

For the venom characterization of *A. geniculata*, the crude venom was analyzed by SDS-PAGE revealing two main bands, the first with 45 kDa and a second with proteins below 10 kDa. Both bands were digested in gel with trypsin to analysis by LC-MS/MS followed by the quantification of the proteins obtained by spectral counting realized by extracted-ion chromatography. The quantification revealed that the most part of these proteins’ present homology with CRISP3, but were also found two metaloendoproteinases, a pancreatic-like triacylglycerol lipase, a carbonic anhydrase, and a hyaluronidase. Authors suggested that the venom proteases major role is the activation of protoxins once them present homology with the proteases that cause activation precursor proteins [39]. 

In a study realized in 2017, Walter *et al*. [40] investigated the correlation between the venom injection and extra-oral digestion using *A. geniculata* model of Theraphosidae. For this purpose, the authors realized an overlap of the venom and digestive fluids proteins, to determine a possible role of the venom in the extra-oral digestion. The digestive fluids were extracted and analyzed by nano flow LC/MS-MS and 36 from 294 proteins were quantified. The overlap with the venom toxins previously described by [39] showed the presence of 11 common proteins from 118 present in the composition of the venom [40].

Wilson *et al*. [41] isolated and characterized two novel polyamines from the venom of multiple Theraphosidae spiders, including *A. geniculata*. Thirty-one species of the Theraphosidae family were selected to an initial cytotoxic evaluation of the crude venom against MCF-7 cells, and 17 of them presented significant activity (considered by authors as over 50% inhibition when compared with the control). From those, 8 venoms were chosen to be fractionated by RP-HPLC, including the one from *A. geniculata* and the resultant fractions were submitted to cytotoxic assays against MCF-7, SK-MEL-28 (ATCC®HTB-72) and NFF cells [41].

All the venoms presented early eluting fractions with activity against MCF-7 cells. The polyamine PA_366_ was isolated from *Phlogius* sp. venom by RP-HPLC. The venoms from the other seven spiders were analyzed by one-dimensional NMR spectroscopy, which confirmed the presence of the molecular mass 366.2573 Da, corresponding to PA_366_ in the venom of *A. geniculata* [41].

The PA_366_ presents an aromatic head group (2-hydroxy-3-(4-hydroxyphenyl)propanal) which is possible correlated with the cytotoxicity showed by the molecule, once this group is the only structural difference between PA_366_ and another polyamine named as PA_389_, which only displayed cytotoxic activity in concentration higher than 1mM, while PA_366_ is active even in concentrations varying from 1 to 10 µM. Authors suggested that PA_366_ may cause paralysis in preys due the similarities with PA_389_. [41].

## Avicularia juruensis

*Avicularia juruensis* (Mello-Leitão 1923; sub-family Aviculariinae), mostly found in Amazonia, is commonly known as Amazonian pink toe spider. They occur in South America (Brazil, Ecuador, Peru, and Colombia) and can be found in tree trunks between 1.5 and 3 m high. Most inhabited trees accommodated single individuals [42, 43].

Ayroza *et al*. [42] fractioned *A. juruensis* crude venom by RP-HPLC, fractioned *A. juruensis* crude venom, and the obtained fractions were used to determine antimicrobial activity by liquid growth inhibition assays for target pathogens. The antimicrobial assay showed the presence of four antimicrobial fractions, which were purified by 11 compounds ranging molecular weight from 3.5 to 4.5 kDa [42].

Juruin is an alternative name to U-theraphotoxin Aju1a, the first Juruen toxin to be completely purified, MALDI-TOF/MS analysis revealed a 4005.83 Da molecular mass and the sequence of 38 amino acids (Table 1) was obtained by *de novo* sequencing. Juruin exhibits three disulfide bonds, between the cysteines 1-4, 2-5 and 3-6, the same array is common to all the toxins from spiders that contain ICK motif [42].

Juruin showed antifungal activity against *Candida* spp*. (*MICs between 2.5 and 5μM), and *Aspergilus niger* (MIC between 5 and 10 μM). However, it did not show activity against *M. luteus*, *S. epidermidis*, *S. aureus*, *E. coli*, *P. aeruginosa*, and *B. bassiana* even at 100 μM. Juruin did not exhibit hemolysis against human erythrocytes in concentrations up to 10 μM [42].

## *Lasiodora* sp.

**Figure 7.**
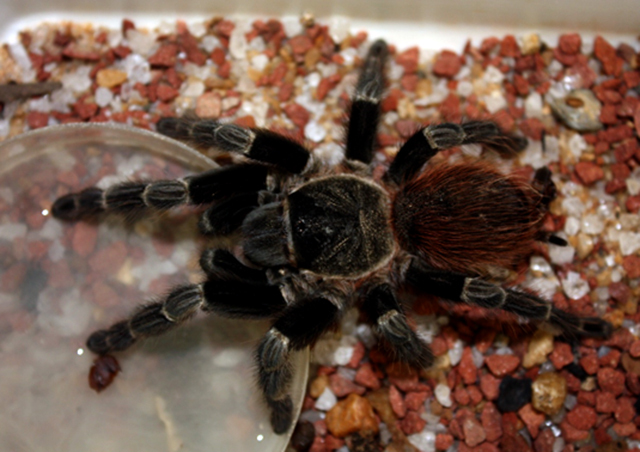
*Lasiodora* sp. collected in Bahia state (Correntina city). Photo by Osmindo R. Pires Jr.

The genus *Lasiodora* (Koch 1850) are referred as tarantula bird-eating spiders or baboon spiders. The 33 known species are distributed among Brazil, Argentina, Uruguay, Bolivia and Costa Rica, with 25 of them occurring only in Brazil [2]. It is generally considered hazardous due to its size and appearance (Fig. 7), but there are no reports of human deaths caused by these species [3]. 

In 2001, Kushmerick *et al*. [44] evaluated the activity of the venom against murine GH3 cells (ATCC® CCL-82.1) Ca^2+^ and Na^+^ channels by whole-cell patch clamp and imaging analysis. The crude and dialyzed venom (400 µg/mL) made the normal oscillations of Ca^2+^ in GH3 cells stop and affected the L-type Ca^2+^ channel by reducing the channel conductance and the intracellular Ca^2+^ in the presence of Na^+^ channels blocked by tetrodotoxin (TTX). The activity does not change in presence of muscarinic receptors blocked by atropine. At last, the experiment was conducted without TTX, however, the venom still affected the Ca^2+^ oscillations, suggesting that the venom also acts in the Na^+^ channels [44]. 

Kalapothakis *et al*. [45] tested the *Lasiodora* venom in the isolated heart of male Wistar rats (*R. norvegicus*). When administered in concentrations varying from 10 to 100 µg, the venom caused a reversible dose dependence response, decreasing the heart rate. The highest dose provoked bradycardia and transient cardiac arrest. The venom effect was potentiated when applied in presence of anticholinesterase, neostigmine or tetrodotoxin; however, vesamicol (drug that act pre synaptically by inhibiting acetylcholine), reduced the effects and atropine completely blocked the venom effects. The authors concluded that the venom activates TTX-resistant Na^+^ channels causing the release of acetylcholine vesicles from parasympathetic terminals [45].

In 2004, Vieira *et al*. [46] described LTx1, LTx2, and LTx3 (Table 1) from a *Lasiodora* sp. venom gland cDNA library. The cDNA library screening was realized with ELISA, whole-venom antisera and PCR techniques. The three lasiotoxins showed significant levels of similarity with HwTX-II from *Selenocosmia huwena*, BsTX from *Brachypelma smithii* and ESTX from *Eurypelma californium*, toxins already described from Theraphosidae spiders [34]. LTx1, LTx2, and LTx3 were further named to U1-theraphotoxin-Lsp1a, U1-theraphotoxin-Lsp1b, U1-theraphotoxin-Lsp1c, respectively (Table 1) [46].

 Another two predicted sequences of toxins presented in *Lasiodora* sp. venom were registered in UniProt Database. Originally known as LTx4 and LTx5, these toxins are now entitled U2-theraphotoxin-Lsp1a and U3-theraphotoxin-Lsp1a (Table 1), under the entries A3F7X1 and A3F7X2 [47].

Dutra *et al*. [48] expressed and pharmacologically characterized LTx2. This toxin was expressed by transformed *E. coli* BL21DE3 and purified by RP-HPLC. Imaging analysis on confocal microscopy was performed to evaluate the LTx2 recombinant activity on Ca^2+^ channels of murine BC3H1 cells (ATCC®CRL-1443) revealing the toxin capacity to completely block L-type Ca^2+^ channels at 80 µM even without the presence of TTX, which provoked the same effect at 1 µM [48].

Soares *et al*. [49] reported in 2011, the purification and characterization of the first serine protease inhibitor extracted from *Lasiodora* sp. hemocytes, which was named EILaH. The hemocyte extract shows activity against trypsin, chymotrypsin, urokinase, tissue plasminogen activator, and human neutrophil elastase, with the last subtract getting 99% of inhibition. EILaH was purified by affinity chromatography (Trypsin-Sepharose column) followed by RP-HPLC, and then analyzed by SDS-PAGE, revealing an 8 kDa protein. MALDI-TOF/MS analysis confirmed the presence of an 8274 Da protein, which was partially sequenced by Edman degradation resulting in the N-terminal sequence LPC(PF)PYQQELTC [48]. The authors also evaluated the antimicrobial activity of both EILaH and *Lasiodora* sp. hemocyte extract against *B. subtilis* (ATCC-6633), *S. aureus* (ATCC-6538), *E. faecalis* (ATCC-6057), *E. coli* (ATCC-25922) and *K. pneumoniae* (ATCC-29665) as seen in Table 2. EILaH only showed activity against *E. faecalis* (MIC = 227.5 µg/mL), while the hemocyte extract presented activity against *B. subtilis* and *E. faecalis* (MICs were not present by authors), indicating the presence of other antimicrobial agents in the hemocytes [49].

Horta *et al*. [4] demonstrated that *Lasiodora* sp. venom caused dose-dependent vasodilatation in male Wistar rats (*R. norvegicus*) aortic rings contracted with phenylephrine (IC_50_ = 6.6 ± 1.8 µg/mL), but only when in contact with a functional endothelium. The venom also caused the Ser^1177^ phosphorylation activating endothelial nitric oxide synthase (NOS) function, which was determined by Western Blot. The active compound present in the venom was isolated using RP-HPLC, analyzed in ESI-MS/MS, which revealed two ions with 348.1 and 136.2 Da, and through NMR it was possible to confirm that these ions correspond to diphosphate adenosine (ADP) and adenosine monophosphate (AMP). The authors suggested that ADP is the main component for the vasodilatation effect caused by the *Lasiodora* sp*.* venom [4].

The description of antimicrobial activity of *Lasiodora* sp*.* crude venom, was realized in 2016 using concentrations ranging from 3.9 to 500 mg/mL [50]. It was observed that the venom is bacteriostatic (more than 50% of inhibition when compared to the control) for *S. aureus*, *P. aeruginosa*, and *K. pneumoniae*; bactericidal (more than 90% of inhibition when compared with the control) to *Aeromonas* sp., *B. subtilis*, and *M. luteus*; fungistatic against *C. tropicalis* and *C. cruzei* and fungicidal against *C. parapsilosis* and *C. albicans*. The activity against human peripheral blood mononuclear cells (PBMC) was also evaluated, which resulted in an induction of apoptosis at 0.1 mg/mL of crude venom, showing that the venom is cytotoxic. However, when tested against *M. musculus* erythrocytes, it demonstrated low hemolytic activity (EC_50_ = 757 mg/mL) [50].

The authors also fractionated the crude venom by RP-HPLC, and fractions were submitted to electrospray *tandem* mass spectrometry with a quadrupole/orthogonal acceleration time-of-flight spectrometer (Q-TOF/MS). They presented homology with the peptides U1-theraphotoxin-Lp1a (lasiotoxin-1), U1-theraphotoxin-Lp1c (lasiotoxin-3), U3-theraphotoxin-Lsp1a (LTx5), and U-theraphotoxin-Asp3a. The mass spectrometry also identified proteins as Phospholipase A2 (PLA_2_) and Hyaluronidase [50].

## Lasiodora parahybana

**Figure 8.**
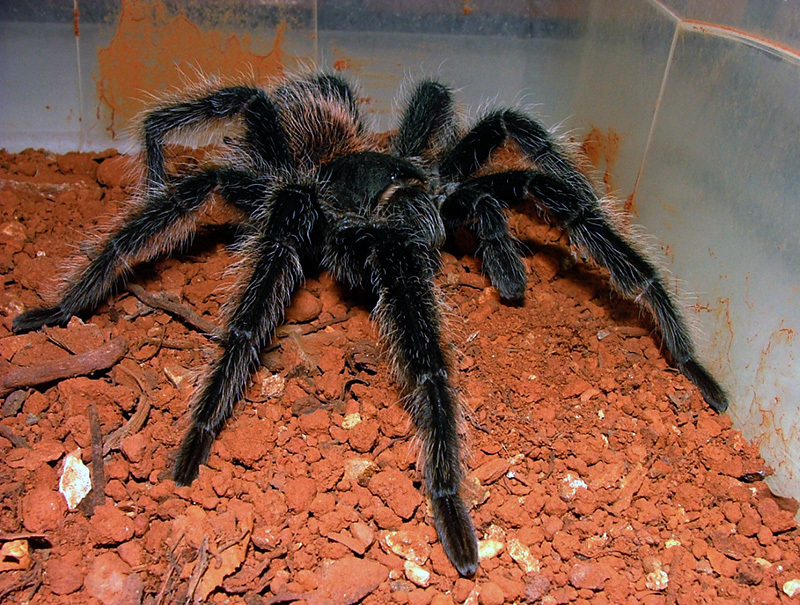
Male specimen of *Lasiodora parahybana*. Collection of Arachnids from the Department of Zoology, University of Brasília, no. 3681. Photo by João de Jesus Martins.

*Lasiodora parahybana* (Mello-Leitão, 1917; Fig. 8) is commonly known as Salmon pink tarantula [51]. This species is endemic to Brazil, occurring in North Eastern region of the country [2, 51].

Escoubas *et al*. [52] described two neurotoxins isolated from *L. parahybana* venom, LpTx1 and LpTx2, purified in two chromatography steps (ion exchange HPLC followed by RP-HPLC). The crude venom toxicity was tested intracerebroventricularly in mice (*M. musculus*) and intrathoracically in crickets (*Gryllus bimaculatus*). Both species presented paralysis followed by death. However, the mice’s first presented symptoms were an increase in motor activity and restlessness with death occurring 40 minutes after the injection. In crickets, both paralysis and death occurred quickly after injection. The fractions with activity were isolated, submitted to reduction, alkylation, and sequenced by Edman degradation. LpTx1 and LpTx2 have 49 amino acids sequences (Table 1) with only two different amino acids. MALDI-TOF/MS analysis revealed molecular masses of 5722 and 5674 Da, respectively. The toxins present high homology (74%) with toxins isolated from the spiders *Eurypelma californicum* (ESTX) and *Brachypelma smithii* (BSTX). To the publication date, molecular targets are still unknown [52].

Currently, the toxins LpTx1 and LpTx2 are registered in Uniprot database as U1-theraphotoxin-Lp1a and U1-theraphotoxin-Lp1b, respectively (Table 1). 

In 2002, Escoubas *et al*. [53] studied sex-linked variations on the venoms of eight species of spiders, including *L. parahybana*, and demonstrated by RP-HPLC that there is no expressive qualitative variation. However, in MALDI-TOF/MS analysis authors identified molecular masses 3106.6 Da and 3535.3 Da present only in female individuals, and molecular masses 3918.1, 7841.9, and 8274.3 Da only in male individuals, showing that quantitative difference exists between sexes, but they concluded that the venom does not show representative variation when compared the sex of *L. parahybana* [53].

Escoubas and Rash [1] made a general comparison of many tarantula venoms. The lasiotoxins presented in *L. parahybana* (LpTx1 and LpTx2, also known as U1-theraphotoxin-Lsp1a and U1-theraphotoxin-Lsp1b, as seen in Table 1) venom have larger sequences (49 amino acids, Table 1) than the average 31-41 amino acid of the peptides extracted from Tarantula’s venom. Lasiotoxins were classified as DDH (disulfide directed β-hairpin) and their primary sequences compared with *Eurypelma* spider toxins (ESTxs) and Huwentoxin-II from *Selenocosmia huwena* (HwTxII), indicating that they assume the same conformation even with the extra 13 amino acids, forming a fourth disulfide bond [1].

The peptide profile of *L. parahybana* venom gland using conventional methods such liquid chromatography coupled to an electrospray-ionisation hybrid quadrupole time of flight mass spectrometer (LC/ESI-QqTOFMS), matrix-assisted laser desorption/ionization time-of-flight (MALDI-TOF/MS) [53]. The analysis of fresh tissue was performed by MALDI-TOF/MS along with venom direct analysis by nanoESI-QqTOF/MS. These experiments resulted in 81 monoisotopic molecular masses ranging from 601.38 to 43499 Da with the molecules 601.38, 729.35, 3846.17, 424.60, 4691.03, 4846.36, 5020.39, and 7759.73 Da been considered representative of the mass fingerprint once they are always presented in the spectra [54].

The authors also compared the venom of juvenile *L. parahybana* (4-years old) and adults (8 and 14 years old). The major difference presented by juvenile to 8 years adult spiders was the presence of a 5723.76 Da ion and absence of a 5642.48 ion. The 14 years adults presented the same molecular masses of the 8 years old but fractions with more intensity. The *in situ* analysis showed differences on the peptide levels in different cells distributed in the gland, suggesting that the compounds are produced by different cell subpopulations. One of these different compounds is an 8668.94 Da molecule found on the top of the gland that was supposed to be a novel non-processed precursor or an enzyme involved in the toxin maturation [54]. 

## Grammostola iheringi

*Grammostola iheringi* (Keyserling, 1891) is a Theraphosidae South American spider. Found in Southern states of Brazil, Northern states of Argentina, Chile, Paraguay, and Uruguay [55]. 

*G. iheringi* venom was studied using a proteomic a bottom-up approach multidimensional protein identification technology (MudPIT) approach [56]. PepExplorer tool was used for bioinformatics analysis comparing proteins based on phylogenetically close organisms, resulting in 395 proteins identified from the venomous extract. Approximately 70% of these proteins were classified as predicted, matching with neurotoxins that act on ion channels, proteases such as serine proteases, cysteine proteinases, metalloproteinases, aspartic proteinases, carboxypeptidases and cysteine-rich secretory enzymes (CRISP), and molecules with unknown targets. The other 30% matched with proteins and enzymes already described in databases. *De novo* sequences showed high similarity with sequences from spiders and scorpions [56]. 

## Grammostola pulchra

*Grammostola pulchra* (Mello-Leitão, 1921), is a species of spider endemic to Brazil known as Brazilian black tarantula, occurring in the states of São Paulo, Paraná, Santa Catarina and Rio Grande do Sul [2, 57]

In 1998, Escoubas *et al*. [58] described a combination of RP-HPLC, capillary electrophoresis and MALDI-TOF/MS in order to create a venom fingerprint of its peptides as an effective method to solve problems as identification of samples (animal or venom source) or evaluation of similarity of spiders based in the venom. Among the animals used to develop this study were two samples of *Grammostola*, one previously identified as *G. pulchra* and a *Grammostola* sp. specimen. MALDI-TOF/MS of both samples presents 25 molecular masses ranging from 3410 to 6855 Da with minor changes of intensity and mass between each other. With the combination of data obtained from the chromatography, mass spectrometry and CZE electrophorograms the authors concluded that both specimens are *G. pulchra* [58].

## Vitalius dubius

*Vitalius dubius* (Mello-Leitão, 1923) is a medium-sized, non-aggressive Theraphosidae found in southeastern Brazil, occurring in the southern part of the Brazilian state of Minas Gerais and in the state of São Paulo [59].

A partial characterization of *V. dubius* venom was performed in 2009 [60]. The venom presented hyaluronidase activity in turbidimetric assay and confirmed by hyaluronic acid SDS-PAGE zymogram. Aliquots up to 300 µg of venom did not show any proteolytic activity against elastase, casein, and collagen [60]. An ELISA test was performed using 0.3 mg/mL of *V. dubius* venom against an IgG purified by affinity from arachnidic antivenom produced from *Phoneutria nigriventer*, *Loxosceles gaucho*, and *Tityus serrulatus*. *V. dubius* venom presented lower cross-reactivity when compared with 0.1 mg/mL of *P. nigriventer and T. serrulatus* venoms. SDS-PAGE electrophoresis (15%) showed molecular masses varying from 6 to 130 kDa, followed by immunoblotting in polyacrylamide gels (10%) showing molecular masses with at least 30 kDa. The venom was also purified by RP-HPLC resulting 13 fractions (described as 5 major and 8 minor fractions) [60].

In 2014, Sutti *et al*. [61] described a hyaluronidase (hyase) purification from the venom using gel filtration and RP-HPLC. The hyaluronidase had 43 kDa mass, obtained by SDS-PAGE analysis. The activity is specific to hyaluronic acid and the optimal conditions for activity were pH between 4 and 5; temperature between 35 and 40°C. The addiction of chondroitin decreased the activity, however, antilonomic, antiophidic, and antiscorpionic serum to hyase did not inhibit its enzymatic activity. However, hyase activity was inhibited by antiaracnidic serum in a dose-dependent manner [61].

A toxin of 728 Da named VdTX-1 was purified by Rocha-e-Silva *et al*. [62] from the venom of *V. dubius*. VdTX-1 showed a neuromuscular activity capable of blocking nicotinic receptor. The toxin was tested in biventer cervicis muscles of male Swiss white mice and male HY-LINE W36 chicks. Authors suggested that *V. dubius* venom contains at least two components that affect neurotransmission in vertebrates. The venom caused progressive neuromuscular blockade, which was reversible by washing, and muscle contracture. Contractures caused by the application of acetylcholine and KCl were attenuated by the venom. VdTX-1 also abolished carbachol-induced depolarizations and blocked nicotinic receptors non-competitively to produce reversible blockade without muscle contracture [62]. 

VdTX-1 has antimicrobial activity described by Sutti *et al*. [63]. The toxin presented activity against multiple fungi and bacteria (Table 1), among *Candida* species, Gram-positive bacteria and two strains of *E. coli* with MICs ranging from 6.25 to 50 µM [63].

Rocha-e-Silva *et al*. [64] described the formation of edemas in male Wistar-Hanover rats (*R. norvegicus*) caused by *V. dubius* crude venom. The venom was applied in dorsal skin or hind paw of the rats provoking dose dependent response, which was measured by plasma extravasation [64].

To elucidate the mechanisms behind this activity, the authors evaluated the plasma extravasation caused by the venom in presence of a pool of receptor antagonists to the potential pathways involved in the formation of edemas: cyproheptadine (2 mg/kg), for both serotonin 5-hydroxytryptamine_1/2_ and histamine H_1_ receptors completely inhibited of the plasma extravasation; indomethacin (10 mg/kg), a nonselective COX inhibitor, the nitric oxide synthase inhibitor L-NAME (100 nm/site) and neurokinin NK_1_ receptors antagonists known as SR140333 (1 nm/site) caused partial abolition of the plasma extravasation; bradykinin B_2_ receptor JE049 (0.6 mg/kg), mepyramine (histamine H_1_ receptor inhibitor, 6 mg/kg), and SR48968 (neurokinin NK_2_ receptor inhibitor 0.3 nm/kg) did not caused any reduction of the plasma extravasation [64].

With these results, the authors suggested that the edema formation caused *V. dubius* venom involves serotonin, COX products and nitric oxide, but does not involve histamine and bradykinin. The neurokinins results indicate the participation of tachykinin mediated by NK_1_ neurokinin receptors [64]. 

## Nhandu chromatus

**Figure 9.**
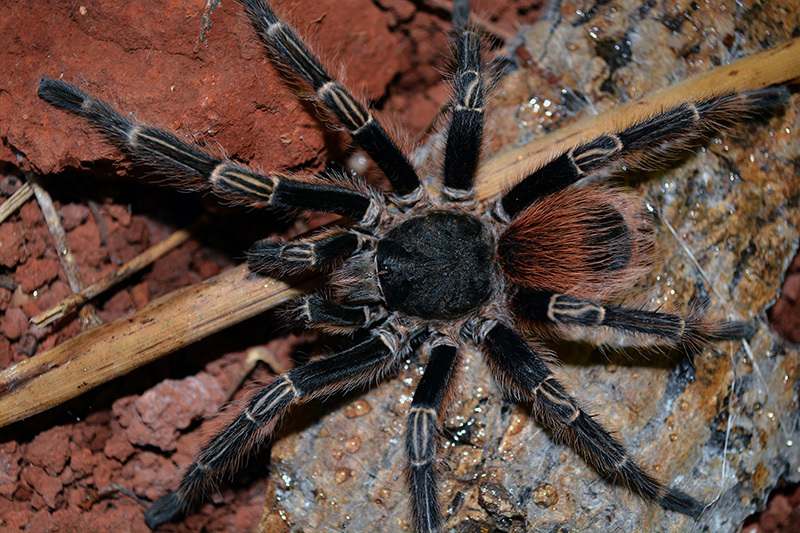
Male specimen of *Nhandu chromatus*. Collection of Arachnids from the Department of Zoology, University of Brasília, no. 8716. Photo by Paulo César Motta.

*Nhandu chromatus* (Schmidt, 2004; Fig. 9) is a Brazilian endemic spider commonly known as red and white tarantula. It is a terrestrial species, usually found in burrows and presents approximately 17 cm of leg spam [2, 65].

Rodríguez-Rios *et al*. [66] described hyaluronidase as a common component of Theraphosidae venom. In 2017, the authors conducted an experimental investigation in the venom of 13 different species including *N. chromatus*, with all presenting hyaluronidase activity in bands varying from 34 to 46 kDa [66].

*N. chromatus* venom was analyzed by tricine-SDS-PAGE followed by a 2D-SDS-PAGE, the low molecular mass compounds were reduced, alkylated and in-gel digested with trypsin. *N. chromatus* showed two bands with 61.8 and 36.8, with the 61.8 kDa band been the only in the entire experiment with mass aside the standard 34-46 kDa. The isolated fractions were analyzed by LC-MS/MS to find hyaluronidase-like compounds. The activity was tested by turbidimetric assay and confirmed by SDS-PAGE and 2D-SDS-PAGE (14%) zymograms with hyaluronic acid [66].

The study performed by Wilson *et al*. [41] revealed cytotoxic activity of *N. chromatus* venom against MCF-7 cells even without the presence of the polyamines PA_366_ and PA_389_, previously described as the primary focus of the study. The concentration utilized to perform the inhibition experiment against MCF-7 and IC_50_ were not show in the paper [41].

## Conclusion

This review aims to highlight the pharmacological potential of chemical compounds from Brazilian Theraphosidae spider venoms. Thanks to the advance of science, poisons and venoms have become a great biotechnological template/tool for drug design, since they are a rich source in bioactive components with the most diverse molecular targets.

Despite the large number of described Theraphosidae spider species, about 185, only 13 of them present any toxinological characterization report of crude venom and/or isolated compounds. Although the Brazilian Theraphosidae venom has a remarkable pharmacological potential, there is scarce research available on it. For a clearer understanding, species/compound/activity is summarized in Table 3.

Among the countless challenges of modern medicine, we highlighted the microorganism resistance to conventional antibiotics, due to the indiscriminate use of them, and in addition, the dramatic decline of new antimicrobials development. A great example of the spiders’ potential for drug discovery are the antimicrobial peptides or “low weight mass compounds” presented here that have shown activity against a broad spectrum of bacteria and fungi.

Gomesin, which was originally reported to have an antimicrobial activity, also demonstrated activity against melanoma cells. Similarly, mygalin, when incorporated into silver nanoparticles, increased its already described antimicrobial activity and also revealed antitumor activity. The discovery of alternatives for cancer therapies is desired, since chemotherapy involves the use of drugs to selectively destroy the tumor or limit its growth. However, the use of these cytotoxic agents has several side effects, such as bone marrow suppression, gastrointestinal lesions, nausea, in addition to the development of clinical resistance.

**Table 3.** Documented activities from Brazilian Theraphosidae venom compounds.

Species	Compound	Activity	Target	Reference
*T. blondi*	Crude venom	Neuromuscular blockage	Mouse phrenic nerve-diaphragm preparation	Fontana *et al.* [14]
*T. blondi*	Crude venom	A-type currents inhibition on recombinant Kv4.2 channels	Recombinant C57/B16 Recombinant HEK 293	Ebbinghaus *et al.* [15]
*T. blondi*	κ-theraphotoxin-Tb1a	Inhibition of recombinant Kv4.2 channels Slowed Kv 4.2 kinetics	Recombinant C57/B16 Recombinant HEK 293	Ebbinghaus *et al.* [15]
*T. apophysys*	TRTX-Tap1a Recombinant TRTX-Tap1a	Inhibitory activity on Na_v_ channels Inhibitory activity on Ca_v_ channels	Recombinant C57/B16 Recombinant HEK 293	Cardoso *et al.* [18]
*T. apophysys*	TRTX-Tap2a Recombinant TRTX-Tap2a	Inhibitory activity on Na_v_ channels Inhibitory activity on Ca_v_ channels	Recombinant C57/B16 Recombinant HEK 293	Cardoso *et al.* [18]
*A. gomesiana*	Venom peptidic fraction	Antimicrobial	Gram-negative Yeasts	Abreu *et al.* [20]
*A. gomesiana*	Gomesin	Antimicrobial	Gram-positive Gram negative Fungi	Silva *et al.* [21]
*A. gomesiana*	Gomesin	Antitumoral	MM96L BRAF mutated cells	Ikonomopoulou *et al.* [23]
*A. gomesiana*	Acanthoscurrin-1 Acanthoscurrin-2	Antimicrobial	Gram-negative Yeasts	Lorenzini *et al.* [24]
*A. gomesiana*	Migalyn	Antimicrobial	*E. coli*	Pereira *et al.* [25]
*A. gomesiana*	Migalyn	Immunomodulatory	C57BL/6 mice splenocytes C57BL/6 mice macrophages	Mafra *et al.* [26]
*A. gomesiana*	Migalyn	Anticonvulsant	*R. norvegicus*	Godoy *et al.* [27]
*A. gomesiana*	MygAgNP1	Antimicrobial Antitumoral	*E. coli* MCF-7	Courrol *et al.* [29]
*A. paulensis*	Ap1a	Reducing of electro stimulation	GF-TTM neurons of *D. melanogaster* GF-DLM neurons of *D. melanogaster*	Mourão *et al.* [31]
*A. natalensis*	Rondonin	Antifungal	*Candida* spp. *Trichosporon* sp.	Riciluca *et al.* [34]
*A. natalensis*	µ-TRTX-An1a	Stimulation of action potential spontaneous firing	DUM neurons of *P. americana*	Rates *et al.* [35]
*A. natalensis*	AnHyal	Hyaluronidasic		Barth *et al.* [36]
*A. natalensis*	U1-TRTX-Agm3a	Antimicrobial Antiviral Antitumoral	*In silico* predictions	Câmara *et al.* [37]
*A. natalensis*	TRTX-Ar CRP family	Antimicrobial Antiviral Antitumoral	*In silico* predictions	Câmara *et al.* [37]
*A. geniculata*	Crude venom	Antitumoral	MCF-7	Wilson *et al.* [41]
*A. geniculata*	PA_366_	Antitumoral	MCF-7	Wilson *et al.* [41]
*A. juruensis*	U-theraphotoxin Aju1a	Antifungal	*Candida* spp.	Ayrosa *et al.* [42]
***Lasiodora* sp.**	Crude venom	Na^+^ channels blockage Ca^2+^ channels blockage	*M. musculus* GH3 cells	Kushmerick *et al.* [44]
***Lasiodora* sp*.***	Crude venom	Heart rate reduction	*R. norvegicus*	Kalapothakis *et al.* [45]
***Lasiodora* sp*.***	U1-theraphotoxin-Lsp1b	L-type Ca^2+^ channels blockage	*M. musculus* BC3H1 cells	Dutra *et al.* [48]
***Lasiodora* sp*.***	EILaH	Serine protease inhibition		Soares *et al.* [49]
***Lasiodora* sp*.***	EILaH	Antimicrobial	*E. faecalis*	Soares *et al.* [49]
***Lasiodora* sp*.***	Hemocytes extract	Antimicrobial	*B. subtilis* *E. faecalis*	Soares *et al.* [49]
***Lasiodora* sp*.***	Crude venom	Vasodilatation in aortic rings	*R. norvegicus*	Horta *et al.* [4]
***Lasiodora* sp*.***	Crude venom	Antimicrobial	Gram-positive Gram negative *Candida* spp.	Ferreira *et al.* [50]
*V. dubius*	Crude venom	Hyaluronidasic		Rocha-e-Silva *et al.* [60]
*V. dubius*	Hyase	Hyaluronidasic		Sutti *et al.* [61]
*V. dubius*	VdTX-1	Nicotinic receptor blockage	Biventer cervicis muscles of HY-LINE W36 chicks Biventer cervicis muscles of *M. musculus*	Rocha-e-Silva *et al.* [62]
*V. dubius*	VdTX-1	Antimicrobial	Gram-positive Gram negative Fungi	Sutti *et al.* [63]
*N. chromatus*	Crude venom	Hyaluronidasic		Rodríguez-Rios *et al.* [66]
*N. chromatus*	Crude venom	Antitumoral	MCF-7	Wilson *et al.* [41]

The ability to selectively inhibit ion channels or block receptors to paralyze prey shown by some venom chemical constituents can also be able to reduce chronic pain, or even be useful for the development of drugs that can help the treatment of neural diseases such as Alzheimer's, Parkinson's and seizures.

Concluding, Brazil is a giant in biodiversity, and spiders are truly natural pharmacological libraries. Both facts must motivate researchers and institutions for further studies in toxinological and conservation fields.

### Abbreviations

2D BN/SDS-PAGE: two-dimensional blue native sodium dodecyl sulfate polyacrylamide gel electrophoresis; 2D-NMR: two-dimensional nuclear magnetic resonance; ADP: adenosine diphosphate; AFP: antifungal protein; AMP: adenosine monophosphate; ATCC: American Type Culture Collection; Ca_v_: voltage-dependent calcium channels; cDNA: complementary desoxyribonucleic acid; CFDA: carboxyfluorescein diacetate assay; CFU: colony forming units; COX: cytochrome-C oxidase; CRISP: cysteine-rich secretory proteins; CRP: cysteine-rich proteins; CZE: capillary zone electrophoresis; DAPI: 4',6-diamidino-2-phenylindole; DDH: disulfide directed β-hairpin; DHR: dihydrorhodamine hydrochloride; DUM: dorsal unpaired median neurons; EC_50_: effective dose for 50%; ELISA: enzyme linked immunosorbent assay; ESI-MS/MS: electrospray ionization tandem mass spectrometry; ESI-MS: electrospray ionization mass spectrometry; GF-DLM: giant fiber dorsal longitudinal motor neurons; GF-TTM: giant fiber tergo trochanteral motor neurons; hNa_v_ channels: human voltage dependent sodium channels; IC_50_: inhibitory concentration to 50% inhibition; IFN-γ: interferon gamma; IgG: immunoglobulin G; IL-1β: interleukin 1 beta; iNOS: inducible nitric oxide synthase; KCl: potassium chloride; K_v_ channels: voltage-dependent potassium channels; LC/ESI-MS: liquid chromatography/electrospray ionization mass spectrometry; LC/ESI-QqTOFMS: liquid chromatography electrospray-ionization hybrid quadrupole time of flight mass spectrometer; LC-MS/MS: liquid chromatography tandem mass spectrometry; LC-MS: liquid chromatography mass spectrometry; LD_50_: lethal dose for 50%; LMMF: low molecular mass fraction; L-NAME: Nv-nitro-L-arginine methyl ester; L-NIL: L-N6-(1-iminoethyl)lysine dihydrochloride; LPS: lipopolysaccharide; MALDI-TOF/MS: matrix-assisted laser desorption/ionization time-of-flight mass spectrometry; mic: minimal inhibitory concentration; MS/MS: tandem mass spectrometry; MTT: microculture tetrazolium test; MudPIT: multidimensional protein identification technology; MygAgNPs: mygalin silver nanoparticles; nanoESI-QqTOFMS: nanoelectrospray ionization/hybrid quadrupole time-of-flight mass spectrometry; NFF: neonatal foreskin fibroblasts; NMDA: N-methyl-D-aspartate; PBMC: peripheral blood mononuclear cells; PCR: polymerase chain reaction; PI: propidium iodide; PLA_2_: phospholipase A2; PTZ: pentylenetetrazole; Q-TOF/MS: quadrupole/orthogonal acceleration time-of-flight mass spectrometry; reverse phase-high performance liquid chromatography; ROS: reactive oxygen species; SDS-PAGE: sodium dodecyl sulfate polyacrylamide gel electrophoresis; Th1: T helper 1; Th2: T helper 2; TNF α: tumor necrosis factor alpha; TRTXs: theraphotoxins; TTX: tetrodotoxin; UDMS^E^: Ultra Definition Mass Spectrometry^E^.
